# Two Antimicrobial Peptides Derived from *Bacillus* and Their Properties

**DOI:** 10.3390/molecules28237899

**Published:** 2023-12-01

**Authors:** Yujia Zhang, Zinuo Meng, Shilong Li, Ting Liu, Juan Song, Jia Li, Xiumin Zhang

**Affiliations:** 1 College of Life Sciences, Hebei University, Baoding 071002, China; 15530769668@163.com (Y.Z.); 15032853163@163.com (Z.M.); lsl19991229@163.com (S.L.); 2 The Laboratory and Pathology Department, The Hospital of 82nd Group Army PLA, Baoding 071001, China; liuting198234@126.com (T.L.); songjuan124@163.com (J.S.); 3 College of Life Sciences, Hebei Agricultural University, Baoding 071001, China; qilan82@126.com; 4 Key Laboratory of Microbial Diversity Research and Application of Hebei Province, Hebei University, Baoding 071002, China; 5 Engineering Laboratory of Microbial Breeding and Preservation of Hebei Province, Hebei University, Baoding 071002, China

**Keywords:** *Bacillus*, antimicrobial peptides, stability, minimum inhibitory concentration (MIC), LC-MS/MS

## Abstract

Growth promotion and disease prevention are important strategies in the modern husbandry industry, and for this reason, antibiotics are widely used as animal feed additives. However, the overuse of antibiotics has led to the serious problem of increasing resistance of pathogenic microorganisms, posing a major threat to the environment and human health. “Limiting antibiotics” and “Banning antibiotics” have become the inevitable trends in the development of the livestock feed industry, so the search for alternative antimicrobial agents has become a top priority. Antimicrobial peptides (AMPs) produced by *Bacillus* spp. have emerged as a promising alternative to antibiotics, due to their broad-spectrum antimicrobial activity against resistant pathogens. In this study, two strains of *Bacillus velezensis* 9-1 and *B. inaquosorum* 76-1 with good antibacterial activity were isolated from commercial feed additives, and the antimicrobial peptides produced by them were purified by ammonium sulfate precipitation, anion exchange chromatography, gel chromatography, and RP-HPLC. Finally, two small molecule peptides, named peptide-I and peptide-II, were obtained from strain 9-1 and 76-1, respectively. The molecular weight and sequences of the peptides were analyzed and identified by LC–MS/MS, which were 988.5706 Da and VFLENVLR, and 1286.6255 Da and FSGSGSGTAFTLR, respectively. The results of an antibacterial activity and stability study showed that the two peptides had good antibacterial activity against *Staphylococcus aureus*, *B. cereus,* and *Salmonella enterica*, and the minimum inhibitory concentrations were 64 μg/mL and 16 μg/mL, 32 μg/mL and 64 μg/mL, and 8 μg/mL and 8 μg/mL, respectively. All of them have good heat, acid, and alkali resistance and protease stability, and can be further developed as feed antibiotic substitutes.

## 1. Introduction

Since the 1950s, during which the United States Food and Drug Administration first approved the addition of antibiotics in feed, antibiotics as feed additives have played a huge role in improving animal growth rate, improving the quality of livestock and poultry products, reducing animal morbidity and mortality, improving feed utilization, and reducing costs [1,2,3]. However, with the long-term use of antibiotics in livestock and poultry breeding, problems such as intestinal flora disorders, reduced disease resistance, increased resistance of pathogenic bacteria, and excessive drug residues in meat and egg products have become more prominent, which have been of wide concern by countries around the world [4,5,6]. Following the ban of all food animal growth-promoting antibiotics by Sweden in 1986 [7], the European Union banned the use of antibiotic growth promoters in animal food production in 2006 and the U.S. Food and Drug Administration imposed restrictions on antibiotic use in animal production in December 2016 [8]. The Ministry of Agriculture and Rural Affairs of our country issued Notice No. 194 in 2019, saying that since 1 January 2020, the addition of antibiotics in feed has been completely banned, and this announcement also indicates the real beginning of “Banning antibiotics” in China. “Banning antibiotics” in feed and “Reducing antibiotics, limiting antibiotics “ in the breeding process have become inevitable trends in the development of the livestock feed industry worldwide [9]. However, this will certainly have a huge impact on the global livestock industry, which may lead to a decline in the production level of meat and egg products and a significant increase in breeding costs. Therefore, the development of antibiotic replacement products and reasonable alternative strategies have become a key issue for the healthy sustainable and green development of animal husbandry.

Previous studies have found that antimicrobial peptides (AMPs) have similar characteristics to antibiotics, such as a broad antibacterial spectrum, it is not easy to induce microbial resistance in them, enhanced host immunity, high safety, etc. They have gained widespread attention as one of the most promising alternative antibiotics [10,11]. Antimicrobial peptides (AMPs) produced by *Bacillus* spp. have emerged as a promising alternative to antibiotics [12]. In this study, the antibacterial active strains of *Bacillus* spp. isolated from commercial feed additives were first screened, and the fermentation time of the active strains were optimized. Then, the antibacterial peptides produced by the active strains were isolated, purified, and identified using an ammonium sulfate precipitation method and various chromatographic and mass spectrometry techniques. Finally, the stability and minimum inhibitory concentration of the antimicrobial peptides were determined. This provided a theoretical basis for the application of the antimicrobial peptide in animal husbandry.

## 2. Results and Discussion

### 2.1. Screening of Bacteriostatic Bacillus spp. Strains

*Staphylococcus aureus* ACCC 10499, *Bacillus cereus* ACCC 04315, and *Salmonella enterica* ACCC 01996 were used as indicator bacteria in this study. The 57 strains of *Bacillus* spp. isolated from commercial feed additives were screened by the standardized agar diffusion method [13], and 33 strains with antibacterial activity were obtained. Strain 76-1 and 9-1 showed good inhibitory effects on *Staphylococcus aureus* ACCC 10499 (Figure S1a), *Bacillus cereus* ACCC 04315 (Figure S1b), and *Salmonella enterica* ACCC 01996 (Figure S1c). A 16S rRNA gene sequence analysis showed that strains 76-1 and 9-1 were *B. inaquosorum* and *B. velezensis*, respectively, which were used to isolate antimicrobial peptides subsequently.

### 2.2. Determination of the Optimal Fermentation Time

**Figure 1:**
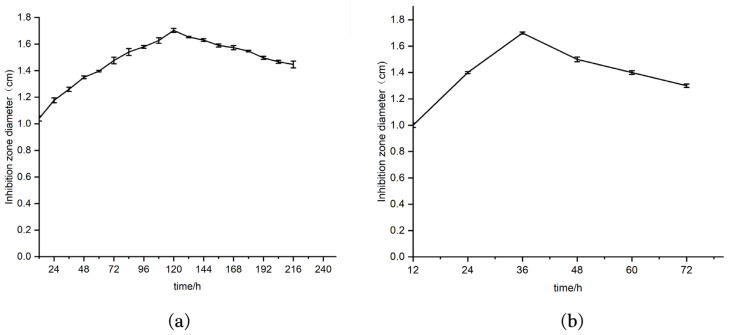
Bacteriostatic activity of strains with different fermentation times. (**a**) Curve of antibacterial activity of strain 9-1 with time; (**b**) curve of antibacterial activity of strain 76-1 with time.

In order to determine the accumulation time of antibacterial substances in active strains, the optimal fermentation times of strains 9-1 and 76-1 were determined, respectively. The antibacterial activity of strain 9-1 gradually increased with time between 12–120 h, and decreased after 120 h (Figure 1a). However, for strain 76-1, the antibacterial activity gradually increased between 12 h and 36 h, reaching the highest level at 36 h, and decreasing after 36 h (Figure 1b). Therefore, 120 h and 36 h were selected as the best fermentation times for strains 9-1 and 76-1, respectively.

**Figure 2:**
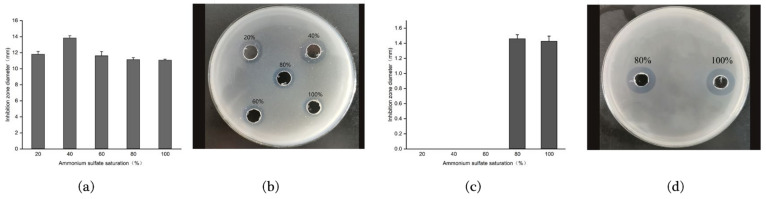
Bacteriostatic activities of fermentation products after precipitation with different concentrations of ammonium sulfate. Graphs (**a**,**c**): bar charts of the bacteriostatic activity of strain 9-1 and 76-1, respectively; (**b**,**d**): inhibition circles of strain 9-1 and 76-1, respectively.

Ammonium sulfate precipitation is one of the most commonly used salting-out methods for antimicrobial peptides purification and has the advantages of safety, mild action conditions, maintaining target protein biological activity, low cost, simple operation, etc. [14]. By investigating the effect of the concentration of ammonium sulfate on the precipitation of antimicrobial peptides, it was found that the precipitation product obtained from the fermentation supernatant of strain 9-1 using a concentration of 20% to 100% ammonium sulfate had antibacterial activity, and the antibacterial circle of the product was the largest when the concentration was 40% (Figure 2a,b), which was selected as the best ammonium sulfate concentration for the purification of antimicrobial peptides of strain 9-1. However, for strain 76-1, only the precipitated products at 80% and 100% ammonium sulfate concentrations showed antibacterial activity (Figure 2c,d). After a comprehensive comparison of factors such as desalt time, purification effect, and cost, 80% was selected as the best ammonium sulfate concentration to precipitate the antibacterial peptide of strain 76-1.

### 2.3. Isolation and Purification of the Antimicrobial Peptides

**Figure 3:**
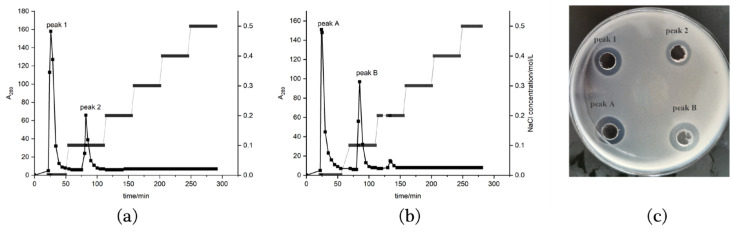
Peak diagram of samples after elution with different NaCl concentrations during DEAE-52 ion exchange chromatography. (**a**): Elution peak diagram of strain 9-1; (**b**): elution peak diagram of strain 76-1; (**c**): inhibition zones against *St. aureus* ACCC 10499 of different elution peak components.

After ammonium sulfate precipitation and desalting, the samples were separated and purified by DEAE Cellulose-52 ion exchange chromatography, and gradient elution was performed with 0–0.5 mol/LNaCl buffer with pH 8.0 0.02 mol/L Tris-HCl. After precipitation with ammonium sulfate and desalting by dialysis, the samples were separated and purified by DEAE Cellulose-52 ion exchange chromatography, and 0.02 mol/L, pH 8.0 Tris-HCl buffer containing 0–0.5 mol/LNaCl was used as gradient eluent. The light absorption value of the eluted samples at 280 nm was detected. Both of the samples from strains 9-1 and 76-1 produced two peaks when the NaCl concentration was 0 and 0.1 mol/L, namely peaks 1 and 2 and peaks A and B, respectively, and the peak shape was basically the same (Figure 3a,b). The activity test showed that the products of peaks 1 and 2, as well as peaks A and B, had obvious antibacterial circles (Figure 3c), indicating that the samples contained antimicrobial peptides.

**Figure 4:**
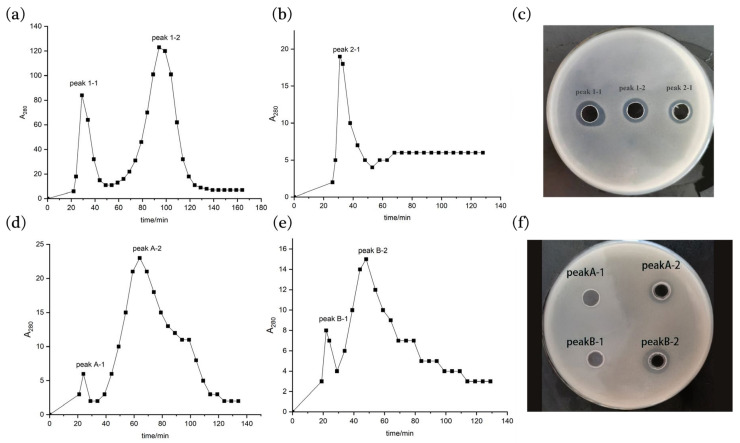
Sephadex G50 gel chromatography purification chromatogram and bacteriostatic activities of crude antimicrobial peptides. Graphs (**a**,**b**): gel chromatogram of ion exchange chromatographic peak 1 and peak 2 of strain 9-1, respectively; (**c**) antibacterial activities of different gel chromatographic peak components of strain 9-1; (**d**,**e**): gel chromatogram of ion exchange chromatographic peak A and peak B of strain 76-1, respectively; (**f**): antibacterial activities of different gel chromatographic peak components of strain 76-1.

Sephadex G50 gel chromatography was used to continue to purify the antimicrobial peptides. Two 280 nm UV absorption peaks 1-1 and 1-2 were obtained by gel chromatography at the DEAE Cellulose-52 ion exchange chromatography peak 1 of strain 9-1 (Figure 4a), and one 280 nm UV absorption peak 2-1 was obtained at peak 2 (Figure 4b), both of which showed antibacterial activities (Figure 4c), but the A280 value of peak 2-1 was significantly lower than that of peak 1-1 and 1-2. After gel chromatography, peak A and peak B of strain 76-1 obtained from DEAE Cellulose-52 ion exchange chromatography each had two A_280_ peaks (Figure 4d,e), and only peak A-2 and peak B-2 were found to have antibacterial activities (Figure 4f).

Finally, the antimicrobial peptides were further purified by anti-phase high-performance liquid chromatography. The peptide from strain 9-1 showed one peak at around 35 min (Figure S2a), while the peptide from strain 76-1 also showed a main peak at around 35 min (Figure S2b). After using the same elution condition to prepare purified antimicrobial peptides samples, it was found that the substances with both of these two peaks had antibacterial activities.

### 2.4. Identification and Property Analysis of Antimicrobial Peptides

**Figure 5:**
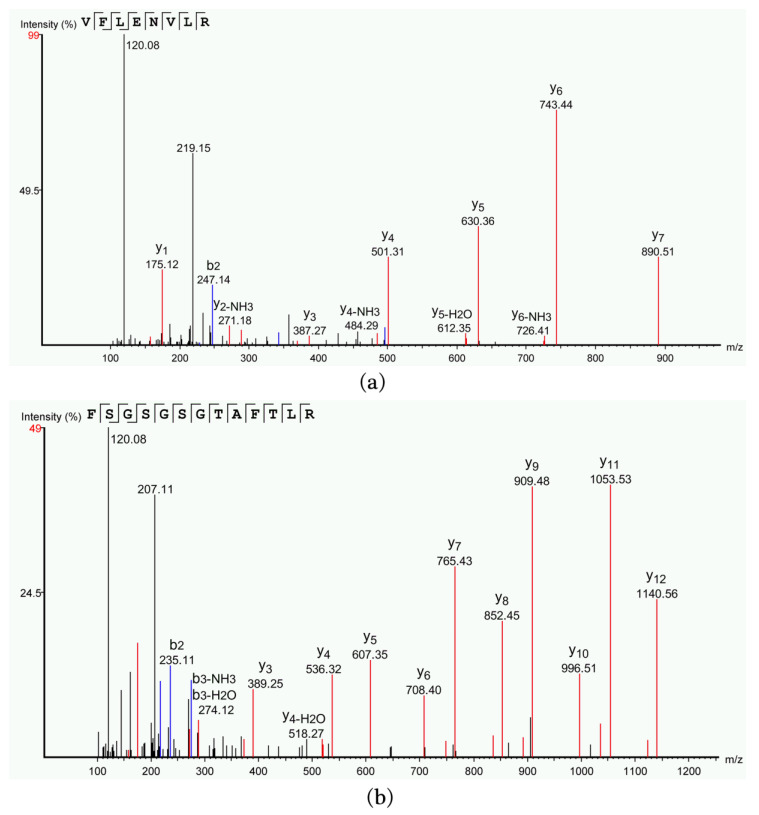
Secondary mass spectrum of antimicrobial peptides. (**a**) Mass spectrum of peptide-I obtained from strain 9-1; (**b**) mass spectrum of peptide-II obtained from strain 76-1.

LC–MS/MS was used to determine and analyze the molecular weight and amino acid sequences of the antimicrobial peptides. The molecular weight of the antimicrobial peptide purified from strain 9-1 is 988.5706 Da and the mass charge ratio is 495.2932 *m*/*z*. The secondary mass spectrum was shown in Figure 5a, which showed that the antimicrobial peptide contained 8 amino acids and the sequence is VFLENVLR. The antimicrobial peptide was named peptide-I. The antimicrobial peptide purified from strain 76-1 has a molecular weight of 1286.6255 Da and a mass charge ratio of 644.3203 *m*/*z*. The secondary mass spectrum was shown in Figure 5b, containing 13 amino acids. The sequence is FSGSGSGTAFTLR and named peptide-II. The amino acid sequences of peptide-I and peptide-II were compared with that of antibacterial peptides reported in the APD database (https://aps.unmc.edu/database, accessed on 22 November 2023), and the highest homologies were only 44.44% and 43.75%, respectively.

Learning about the physicochemical properties of antimicrobial peptides can provide a theoretical basis for their practical production and application. Therefore, we determined the resistance of antimicrobial peptides to temperature and pH protease and the minimum inhibitory concentration.

**Figure 6:**
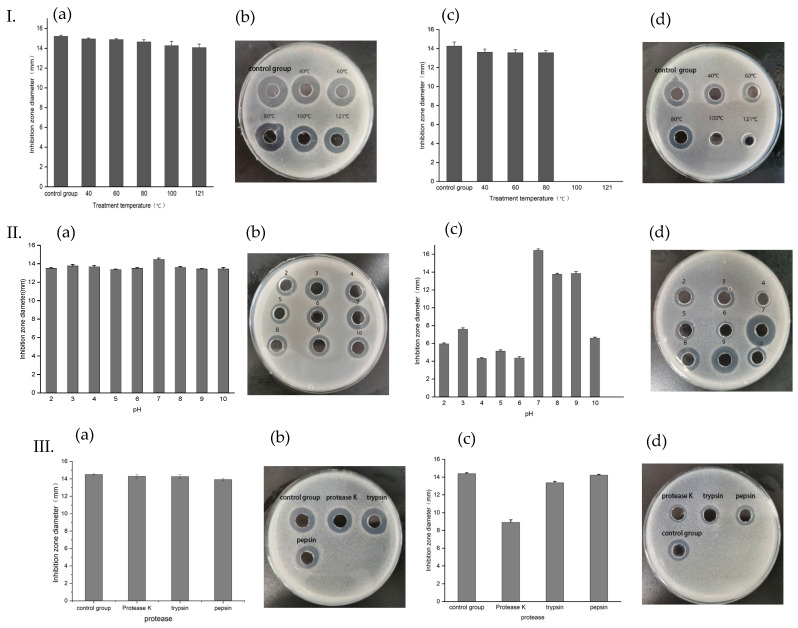
Resistance of the peptides to temperature (**I**), pH (**II**), and proteases (**III**). I: Temperature sensitivity of antimicrobial peptides. Graphs (**a**,**c**): bar chart of temperature sensitivity of peptide-1 and peptide-II, respectively; (**b**,**d**): inhibitory circles of peptide-I and peptide-II against *St. aureus* ACCC 10499 after treatment at different temperatures, respectively. II: pH stability of antimicrobial peptides. Graphs (**a**,**c**): bar chart of pH stability of peptide-1 and peptide-II, respectively; (**b**,**d**): inhibitory circles of peptide-I and peptide-II against *St. aureus* ACCC 10499 after treatment at a different pH. III: Protease stability of antimicrobial peptides. Graphs (**a**,**c**): bar chart of protease stability of peptide-1 and peptide-II, respectively; (**b**,**d**): inhibitory circles of peptide-I peptide-II against *St. aureus* ACCC 10499 after treatment at different proteases, respectively.

In terms of temperature tolerance, the activity of antimicrobial peptide-I did not change significantly with that of untreated samples (Figure 6I(a,b)), that is, the antibacterial activity did not decrease significantly after high temperature treatment, indicating that the antimicrobial peptide had good thermal stability. However, the activity of antimicrobial peptide-II decreased slightly at 40–80 °C, and there was no antibacterial activity after being treated at 100 °C and 121 °C (Figure 6I(c,d)), indicating that the antimicrobial peptide could not tolerate high temperatures. In terms of pH stability, peptide-I could maintain activity at pH 2.0~10.0, and there is no significant difference in its activity at a different pH (Figure 6II(a,b)). Although peptide-II is also active at pH 2.0 to pH 10.0, it is the most active at pH 7.0 (Figure 6II(c,d)). From the stability of protease, the antimicrobial peptide-I was insensitive to protease K, trypsin, and pepsin (Figure 6III(a,b)). The activity of antimicrobial peptide-II was significantly reduced after treatment with protease K, while trypsin and pepsin had no significant effect on its activity (Figure 6III(c,d)).

Lastly, the minimum inhibitory concentrations (MIC) of antimicrobial peptides showed that the MIC of peptide-I against *St. aureus* ACCC 10499, *B. cereus* ACCC 04315, and *Sa. enterica* ACCC 01996 were 64 μg/mL, 32 μg/mL, and 8 μg/mL, respectively. The MIC of peptide-II against *St. aureus* ACCC 10499, *B. cereus* ACCC 04315, and *Sa. enterica* ACCC 01996 were 16 μg/mL, 64 μg/mL, and 16 μg/mL, respectively (Table 1). It can be seen that the antibacterial peptide-I has the lowest MIC and the best antibacterial activity against *Sa. enterica* ACCC 01996, while the antibacterial peptide-II has the same antibacterial activity against *St. aureus* ACCC 10499 and *Sa. enterica* ACCC 01996, with an MIC of 16 μg/mL.

## 3. Materials and Methods

### 3.1. Screening of the Antimicrobial Active Bacillus sp.

A total of 57 strains of *Bacillus* spp. isolated from commercially available feed additives stored in the laboratory were inoculated into NB liquid medium, oscillated at 220 rpm at 30 °C for 20 h, and then inoculated into fermentation medium (glucose 10.0 g, peptone 10.0 g, Na_2_HPO_4_·12H_2_O 2.0 g, NaH_2_PO_4_·2H_2_O 2.0 g, MgSO_4_·7H_2_O 0.5 g, CaCl_2_·2H_2_O 0.2 g, distilled water 1000 mL, pH 7.0–7.2, 121 °C high temperature autoclave sterilization for 15 min) at 1% inoculation rate, and continued to cultivate for 24 h. The culture was centrifuged at 4 °C and 10,000 rpm for 10 min, and the supernatant was stored at 4 °C.

The antibacterial activities of the strains were determined by the agar diffusion method [13]. *Staphylococcus aureus* ACCC 10499, *Bacillus cereus* ACCC 04315, and *Salmonella enterica* ACCC 01996 were inoculated in NB medium, respectively, and oscillated for 16–20 h at 220 rpm, 37 °C. After the concentration of bacterial solution was adjusted to OD_600_ 0.6~0.8, an appropriate amount of bacterial solution was mixed with about 50 °C NA medium at a ratio of 1:50 and poured into sterile plates. The plates after solidification were drilled respectively, into which 50 μL of *Bacillus* spp. strain fermentation supernatant were added and cultured for 12–14 h at 37 °C to observe whether there were bacteriostatic zones. Three parallel experiments were set for each group. The strains with larger antibacterial zones and a wider antibacterial spectrum were selected as the active strains for isolating antimicrobial peptides.

### 3.2. Optimization of the Production Conditions for Antimicrobial Peptides

#### 3.2.1. Determination of the Optimal Fermentation Time

Strains 9-1 and 76-1 with good activities were selected for fermentation. In order to compare the effect of fermentation time on the production of antimicrobial peptides, the fermentation broths were taken at intervals of 12 h and their antibacterial activities against *St. aureus* ACCC 10499 were measured. Three parallel experiments were set up in each group.

#### 3.2.2. Identification of the Optimal Ammonium Sulfate Saturation

The fermentation supernatant of each strain was divided into 5 parts of 50 mL and added into ammonium sulfate with saturations of 20%, 40%, 60%, 80%, and 100%, respectively, and placed in the refrigerator at 4 °C overnight. The solutions with different saturations were centrifuged at 4 °C and 10,000 rpm for 30 min, the supernatant was discarded, and the precipitation was retained as the crude antimicrobial peptide.

### 3.3. Antibacterial Peptide Purification

#### 3.3.1. Dialysis Desalting

Ammonium sulfate in the obtained crude antimicrobial peptides is generally removed by dialysis or molecular rejection [15]. Dialysis was selected for desalting in this study. The precipitate obtained in the ammonium sulfates above was dissolved in sterile redistilled water and packed into a dialysis bag with the retained molecular weight of 3.5 kDa. Then, dialysis was conducted in the refrigerator at 4 °C with sterile redistilled water for 48 h, and the dialyzed liquid was vacuum freeze-dried to obtain lyophilized powder. The solution was dissolved with 0.02 mol/L pH 8.0 Tris-HCl buffer. *St. aureus* ACCC 10499 was used as indicator bacterium and the antibacterial activity was detected by the agar diffusion method [13]. Lastly, the optimal ammonium sulfate saturation was determined by comparing the size of antibacterial zones.

#### 3.3.2. DEAE Cellulose-52 ion-Exchange Chromatography

Ion exchange chromatography is a method of protein separation based on the difference of protein charge. DEAE Cellulose-52 was selected as the filler and balanced with 0.02 mol/L pH 8.0 Tris-HCl buffer at a flow rate of 1 mL/min in this study. The freeze-dried samples of the desalted dialysate were dissolved with 0.02 mol/L pH 8.0 Tris-HCl buffer and then configured into a solution with a concentration of 20 mg/mL. The solution was centrifuged at 10,000 rpm for 10 min, and the supernatant was taken for loading. The loading amount was 2 mL, and the loading time was recorded. After all the samples flowed into the column, the 0.02 mol/L pH 8.0 Tris-HCl buffer containing 0–0.5 mol/L NaCl was used for gradient elution and the eluent was collected. Then, the elution curve was drawn according to the change in the light absorption value at 280 nm. Finally, the antibacterial activity of each peak of the elution curve was detected, and the appropriate eluent concentration was selected according to the antibacterial activity.

#### 3.3.3. Sephadex G50 Gel Chromatography

Sephadex G50 gel chromatography is based on the molecular size of antimicrobial peptides for purification and can be used to desalt samples after ion exchange chromatography [15,16]. The packing Sephadex G50 was used and 0.02 mol/L pH 8.0 Tris-HCl buffer was selected for balancing. The flow rate was adjusted to 0.5 mL/min during the balancing process. For the eluting peaks of samples with antibacterial activity in the previous step, vacuum freeze drying was carried out, and the sample was dissolved by the method of Section 3.2.2. The samples’ loading amounts were 5% of the column volume, and the samples’ loading times were recorded. After the samples were completely flowing into the column, the 0–0.5 mol/L NaCl of pH 8.0 0.02 mol/L Tris-HCl buffer was added for gradient elution with the elution rate of 0.5 mL/min, and the light absorption values at 280 nm were measured. The eluent was collected every 5 min to draw an elution curve according to the change in A_280_ values, and the antibacterial activity of the eluent with the peak value was measured.

#### 3.3.4. Anti-Phase High-Performance Liquid Chromatography (RP-HPLC) Purification and Preparation

RP-HPLC can isolate, purify, and prepare antimicrobial peptides based on their unique physicochemical properties and different retention times [17,18]. The active components after purification in Sephadex G50 gel chromatography were freeze dried by vacuum, from which the dried samples were dissolved with methanol and filtered. C18 column (4.6 mm × 250 mm) was used for HPLC analysis. The loading volume was 10 μL, acetonitrile and water were used as mobile phases for gradient elution in different proportions, the flow rate was 1 mL/min, the column temperature was 30 °C, and the detection wavelength was 254 nm. The HPLC gradient elution procedures were shown in Table 2. By observing the time of the peaks’ occurrence, a suitable gradient elution program was selected and the antimicrobial peptides were prepared by preparative high-performance liquid chromatography. The activities of the antimicrobial peptides were determined by the filter paper disk agar diffusion method [19,20].

### 3.4. Identification and Property Analysis of Antimicrobial Peptides

#### 3.4.1. LC–MS/MS Analysis

In order to determine the molecular weight and sequence of antimicrobial peptides, the prepared antimicrobial peptide samples were sent to Sangon Biotech Co., Ltd. (Shanghai, China) for mass spectrometry identification and analysis by LC–MS/MS. The detailed process was as follows:

The polypeptide samples were dissolved in a washing buffer (0.1% formic acid, 2% acetonitrile), and transferred into a 10 KD ultrafiltration centrifuge tube to centrifugate at 12,000× *g* for 10 min. The solution after ultrafiltration was desalinized using a C18 peptide desalting column (Acclaim PepMap RSLC, 75 μm × 25 cm C18-2 μm 100 Å). The sample was then eluted with an elution buffer (0.1% formic acid, 60% acetonitrile) and the elution solution was transferred to a new Eppendorf tube. The elution samples were centrifugally concentrated and dried, and then redissolved in 100 μL Nano-LC mobile phase A (0.1% formic acid/ultrapure water), then samples were taken and online LC–MS analysis was performed. The dissolved samples were loaded onto a nanoViper C18 column (3 μm, 100 Å) at a volume of 2 μL and then rinsed for desalting at a volume of 20 μL mobile phase (0.1% formic acid/ultrapure water) using an Easy nLC 1200 liquid phase system (Thermo Fisher, Waltham, MA, USA). The samples that were retained on the nanoViper C18 column after desalting were then separated by C18 reverse-phase analysis column (Acclaim PepMap RSLC, 75 μm × 25 cm C18-2 μm 100 Å). The gradient used was an increase in mobile phase B (80% acetonitrile, 0.1% formic acid) from 5% to 38% within 30 min.

Mass spectrometry was performed by Thermo Fisher Q Exactive system (Thermo Fisher, USA) combined with Nanospray Flex™ Ion Sources (Thermo Fisher, USA). The spray voltage was 1.9 kV and the heating temperature of the ion transport tube was 275 °C. The scanning mode of the mass spectrometry was Data Dependent Analysis (DDA), the scanning resolution of the primary mass spectrometry was 70,000, the scanning range was 100–1500 *m*/*z*, and the maximum injection time was 100 ms. A maximum of 20 secondary spectra with charges of 1+ to 3+ were collected in each DDA cycle, and the maximum implantation time of secondary mass spectrometry ions was 50 ms. The impact chamber energy (High Energy Collision Dissociation, HCD) was set to 28 eV for all precursor ions, and the dynamic exclusion was set to 6 s.

The original raw files collected by mass spectrometry were processed and analyzed using PEAKS Studio 8.5 (Bioinformatics Solutions Inc. Waterloo, ON, Canada) software. The database was *Bacillus subtilis* species Protein database downloaded from Uniprot (https://www.uniprot.org/, accessed on 10 May 2023). The search parameters were set as follows: the primary mass spectrometry mass tolerance was 10 ppm, and the secondary mass spectrometry was 0.05 Da. In the processing, Oxidation (M), Acetylation (Protein N-term), Deamidation (NQ), Pyro-glu from E, and Pyro-glu from Q were selected as variable modifications.

#### 3.4.2. Determination of Antimicrobial Peptide Stability

Appropriate amounts of purified antimicrobial peptide samples were placed in a water bath at 40 °C, 60 °C, 80 °C, 90 °C, and 100 °C for 30 min and high-temperature autoclave sterilization at 121 °C for 15 min respectively, to carry out temperature sensitivity tests. *St. aureus* ACCC 10499 was used as an indicator bacterium, and untreated samples were used as controls, with three parallel groups for each sample to observe the change in antibacterial activity. Similarly, the pH of the same amount for the antimicrobial peptide samples was adjusted to 2~10 in intervals of 1, and the neutral pH was 7.0 as the control. The samples with a different pH were kept in a water bath at 37 °C for 30 min. *St. aureus* ACCC 10499 was used as an indicator bacterium to detect the resistance of the antimicrobial peptide to an acid and base. In addition, appropriate antimicrobial peptide samples were mixed with 1 mg/mL solution of protease K, trypsin, and pepsin at 1:1, and were kept in a water bath at 37 °C for 1 h, followed by a boiling water bath for 5 min to inactivate the enzymes. The samples without protease treatment were used as controls and the changes in antibacterial activity were observed.

#### 3.4.3. The Minimum Inhibitory Concentration

The minimum inhibitory concentrations (MICs) of purified antimicrobial peptides against *St. aureus* ACCC 10499, *B. cereus* ACCC 04315, and *Sa. enterica* ACCC 01996 were determined by the double dilution method [21,22]. The indicator bacteria cultures were diluted to an OD_600_ of 0.5 respectively, of which 50 μL of them were added into the sterile 96-well plate, and then 50 μL antimicrobial peptide samples with different concentrations were mixed with each indicator bacterium at a 1:1 ratio. The experiment was performed in three parallel sets. After incubation at 37 °C for 12–18 h, the OD_600_ value was determined by an Automatic growth curve analyzer (Bioscreen C, Turku, Finland), and the minimum concentration of 100% inhibition of indicator bacteria growth was used as the minimum inhibitory concentration [23].

## 4. Conclusions

The present work successfully isolated, purified, and characterized two antimicrobial peptides with good activity. Peptide-I had good resistance to temperature, pH, and proteases and might be desirable for the livestock industry. Peptide-II could bear residues titrating above pH 7.0. In the future, more studies need to be performed, such as testing specificity to pathogens in livestock and poultry and the mechanism of action, toxicity, environmental impact assessment, etc. Moreover, structural improvements to the peptides are still needed to optimize and promise their use. In addition, the large-scale production/purification of the two antibacterial peptides may help us to understand their efficacy in disease prevention in animal husbandry.

## Figures and Tables

**Table 1:** The minimum inhibitory concentration (MIC) of antimicrobial peptides for each indicator bacterium.

Indicator Bacteria	MIC (μg/mL)	
	Peptide-I	Peptide-II
*St. aureus* ACCC 10499	64	16
*B. cereus* ACCC 04315	32	64
*Sa. enterica* ACCC 01996	8	16

**Table 2:** HPLC gradient elution procedures.

Time (min)	Strain 9-1		Time (min)	Strain 76-1	
	Mobile Phase A Acetonitrile	Mobile Phase B Ultrapure Water		Mobile Phase A Acetonitrile	Mobile Phase B Ultrapure Water
0–30	30~70%	70~30%	0–60	40~100%	60~0
30–50	70~80%	30~20%	-	-	-
50–70	80~100%	20~0	-	-	-
70–80	100%	0	-	-	-

## Data Availability

Data are contained within the article and Supplementary Materials.

## Supplementary Materials

The following supporting information can be downloaded at: https://www.mdpi.com/article/10.3390/molecules28237899/s1. Figure S1. Antibacterial activity of primary screening strains against *Staphylococcus aureus* (**a**), *Bacillus cereus* (**b**), and *Salmonella enterica* (**c**). Figure S2. RP-HPLC chromatogram of antimicrobial peptide components. (**a**) Antimicrobial peptide components of strain 9-1; (**b**) Antimicrobial peptide components of strain 76-1.
