# Supplementary material for: Two Antimicrobial Peptides Derived from Bacillus and Their Properties
Source: Molecules. 2023 Dec 1;28(23):7899. doi: 10.3390/molecules28237899 (PMC10708539; doi:10.3390/molecules28237899)
Supplement: Supplementary file 1 [file molecules-28-07899-s001.zip › molecules-2713708-supplementary.pdf]

molecules

MDPI

Supplementary Materials

Two Antimicrobial Peptides Derived from *Bacillus* and Their Properties

Yujia Zhang [1], Zinuo Meng [1], Shilong Li [1], Ting Liu [2], Juan Song [2], Jia Li [3] and Xiumin Zhang [1,4,5,*]

[1] College of Life Sciences, Hebei University, Baoding 071002, China; 15530769668@163.com (Y.Z.); 15032853163@163.com (Z.M.); lsl19991229@163.com (S.L.)
[2] The Laboratory and Pathology Department, The 82nd Military Hospital of PLA, Baoding 071001, China; liuting198234@126.com (T.L.); songjuan124@163.com (J.S.)
[3] College of Life Sciences, Hebei Agricultural University, Baoding 071001, China; qilan82@126.com
[4] Key Laboratory of Microbial Diversity Research and Application of Hebei Province, Hebei University, Baoding 071002, China
[5] Engineering Laboratory of Microbial Breeding and Preservation of Hebei Province, Hebei University, Baoding 071002, China
* Correspondence: zhxiumin1106@126.com

(**a**) (**b**) (**c**)

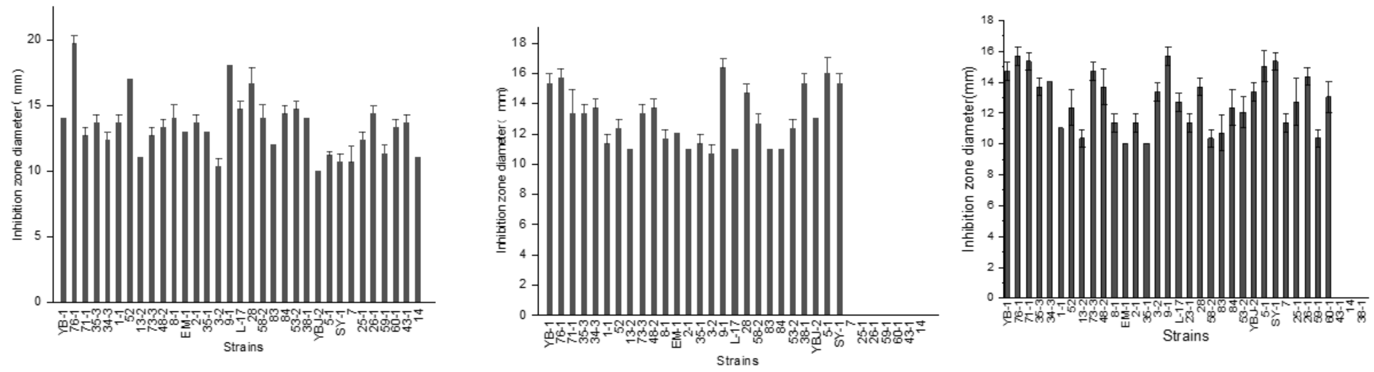

Figure S1. Antibacterial activity of primary screening strains against *Staphylococcus aureus*(**a**), *Bacillus cereus* (**b**), and *Salmonella enterica*(**c**).

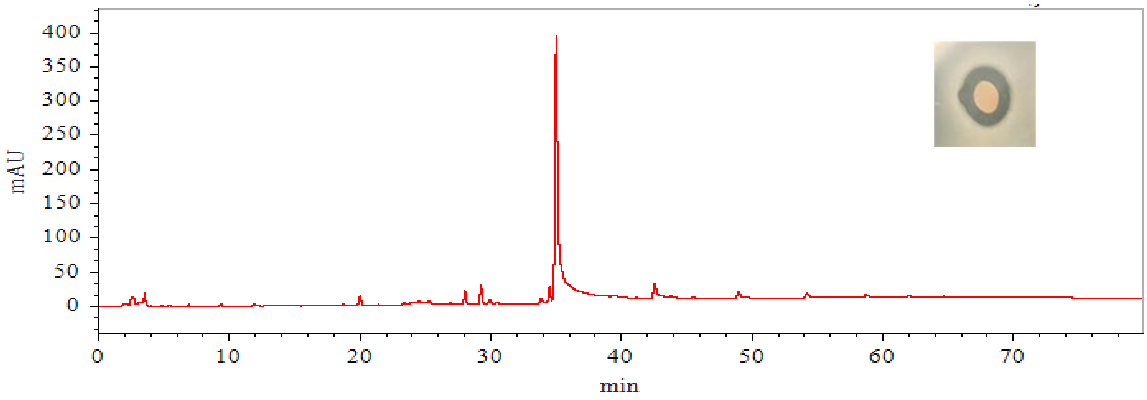

(**a**)

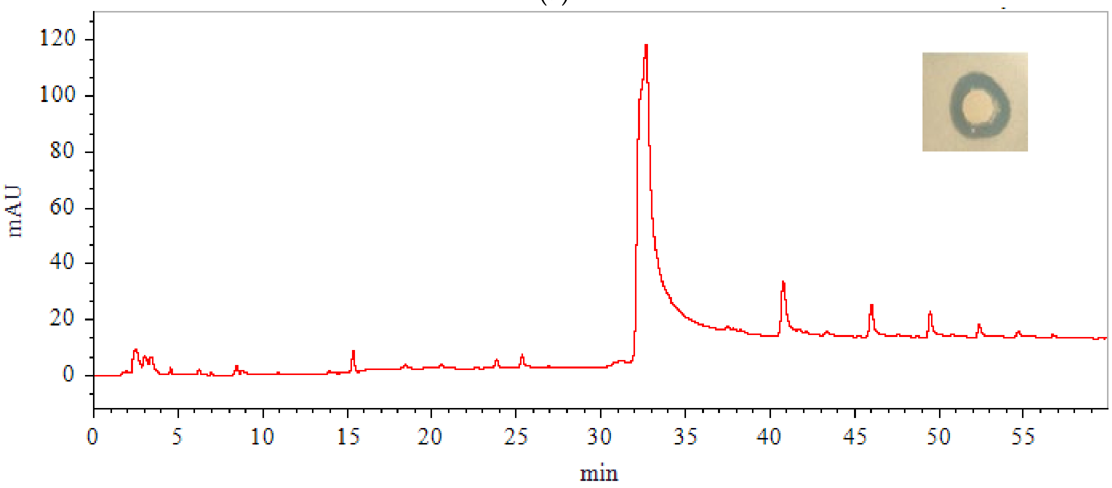

(**b**)

Figure S2. RP-HPLC chromatogram of antimicrobial peptide components. (**a**) Antimicrobial peptide components of strain 9-1; (**b**) Antimicrobial peptide components of strain 76-1.
